# Leadership and organizational change for implementation (LOCI): a randomized mixed method pilot study of a leadership and organization development intervention for evidence-based practice implementation

**DOI:** 10.1186/s13012-014-0192-y

**Published:** 2015-01-16

**Authors:** Gregory A Aarons, Mark G Ehrhart, Lauren R Farahnak, Michael S Hurlburt

**Affiliations:** Department of Psychiatry, University of California, La Jolla, San Diego, CA USA; Child and Adolescent Services Research Center, San Diego, CA USA; Department of Psychology, San Diego State University, San Diego, CA USA; School of Social Work, University of Southern California, Los Angeles, CA USA; Center for Organizational Research on Implementation and Leadership, San Diego, CA USA

**Keywords:** Leadership, Organization, Evidence-based practice, Organizational development, Organizational culture, Organizational climate

## Abstract

**Background:**

Leadership is important in the implementation of innovation in business, health, and allied health care settings. Yet there is a need for empirically validated organizational interventions for coordinated leadership and organizational development strategies to facilitate effective evidence-based practice (EBP) implementation. This paper describes the initial feasibility, acceptability, and perceived utility of the Leadership and Organizational Change for Implementation (LOCI) intervention. A transdisciplinary team of investigators and community stakeholders worked together to develop and test a leadership and organizational strategy to promote effective leadership for implementing EBPs.

**Methods:**

Participants were 12 mental health service team leaders and their staff (*n* = 100) from three different agencies that provide mental health services to children and families in California, USA. Supervisors were randomly assigned to the 6-month LOCI intervention or to a two-session leadership webinar control condition provided by a well-known leadership training organization. We utilized mixed methods with quantitative surveys and qualitative data collected via surveys and a focus group with LOCI trainees.

**Results:**

Quantitative and qualitative analyses support the LOCI training and organizational strategy intervention in regard to feasibility, acceptability, and perceived utility, as well as impact on leader and supervisee-rated outcomes.

**Conclusions:**

The LOCI leadership and organizational change for implementation intervention is a feasible and acceptable strategy that has utility to improve staff-rated leadership for EBP implementation. Further studies are needed to conduct rigorous tests of the proximal and distal impacts of LOCI on leader behaviors, implementation leadership, organizational context, and implementation outcomes. The results of this study suggest that LOCI may be a viable strategy to support organizations in preparing for the implementation and sustainment of EBP.

**Electronic supplementary material:**

The online version of this article (doi:10.1186/s13012-014-0192-y) contains supplementary material, which is available to authorized users.

## Background

The implementation of evidence-based practices (EBPs) [1,2] is important for health and allied health organizations and providers [3]. Leaders can impact the capacity to foster change and innovation [4-7], and the role of “first-level” leaders—those who supervise individuals providing direct services—is particularly critical to organizational effectiveness [8]. First-level leaders are in a position to facilitate EBP implementation [9] and may often be promoted based on clinical expertise with little support or training in effective leadership of workplace change efforts such as EBP implementation. Further, organizational structures and processes can be developed to support first-level leaders in EBP implementation initiatives. In this study, we describe the results of a pilot study of the Leadership and Organizational Change for Implementation (LOCI) intervention, designed to improve leadership and organizational supports to facilitate the implementation and sustainment of EBPs.

### Leadership in health and allied services

First-level leadership is important in health and allied health services. For example, Corrigan and colleagues [10] found a positive association between allied health program leadership and client satisfaction and quality of life. Stronger transformational leadership has been associated with positive work attitudes in both for-profit and non-profit organizations [11-14]. More positive leadership in human service organizations is associated with higher staff organizational commitment [15]. Positive unit level leadership is associated with positive organizational climate, which, in turn, is associated with more positive clinician ratings of provider-client alliance [16]. Effective leadership also supports implementation of task-shifting in surgical units [17]. Finally, more positive first-level leadership is associated with more positive provider attitudes toward adopting EBPs [18].

### Leadership and implementation

Empirical evidence supports the importance of the role of leaders in the implementation process [19-22]. Studies of surgical teams have demonstrated that effective leadership can set the stage for positive team functioning and psychological safety and inclusion that facilitates effective implementation and sustainment of innovative health care procedures [23-25]. Effective leadership supports implementation of person-centered care in nursing homes [26] and hand hygiene in hospital settings [27]. Transformational leadership is important for developing a climate for innovation and positive attitudes toward EBP during large-scale implementation [28]. Reviews and observational studies in nursing have supported the role of leadership in promotion of EBPs [29] and influencing the use of practice guidelines [30]. One mixed method randomized trial found that “relations-oriented” leadership and organizational management processes such as auditing and feedback/reminders supported evidence-based guideline use [31]. Although varying conceptualizations of leadership were utilized in these studies, the Full-Range Leadership (FRL) model [32,33] encompasses a number of leadership styles invoked in these studies, including attending to relationships and attention to performance standards. Although some of these studies included multiple leadership levels, several focused specifically on first-level leadership, as does the current study [19,23-25,28,31].

### Full-Range Leadership

The LOCI training utilized the FRL model to facilitate the development of general leadership and strategic leadership to support EBP implementation and sustainment [34,35]. The FRL model is the most comprehensively researched and validated approach to leadership for individual and organizational development [14,36] and describes leadership behaviors within two primary dimensions: transformational and transactional leadership. Transformational leadership is the degree to which a leader can inspire and motivate others to follow an ideal or a particular course of action [37]. Transformational leadership is comprised of four factors associated with effective organizational functioning [32]: individualized consideration (appreciation of each staff member’s individual contributions and needs), intellectual stimulation (ability to stimulate thinking and accept different perspectives), inspirational motivation (ability to inspire and motivate staff), and idealized influence (degree to which the leader acts confidently, instills pride and respect, and instills values, beliefs, a strong sense of purpose, and collective sense of mission). Transactional leadership focuses on managing incentives and rewards (contingent reward) and meeting quality standards. Both transformational and transactional leadership impact whether and how supervisees accept the vision and direction of the leader and perform assigned job roles and tasks, and both are important for managing and supporting organizational change [36].

The FRL model encompasses leader characteristics identified as important for facilitating EBP implementation in a recent review of nursing leadership and EBP [29]. For example, transactional leadership in the FRL model focuses on providing the support that staff need to complete their daily tasks. Individualized consideration includes several of the behaviors discussed in the review, including providing feedback, encouragement, and consistent communication. This dimension also includes role modeling and being accessible and visible to staff. A leader must also be knowledgeable about EBP to engage in intellectual stimulation with her/his team. Inspirational motivation indicates a leader who can engender enthusiasm for the team’s mission such as EBP use. A leader enacting idealized influence has credibility with his or her team and has obtained their engagement in the team’s goals. LOCI integrates FRL leadership with the goal of increasing leader readiness and support for EBP that may be important in the implementation process [38]. Because of the strong relationships found for FRL behaviors and organizational change effectiveness [36,37], leaders enacting such behaviors should be able to communicate greater support and readiness as well as demonstrating knowledge and perseverance for strategic initiatives such as EBP implementation [38].

Still, it is unlikely that leadership alone will be effective for EBP implementation without attention to the organizational context for change [9] and characteristics of leaders and organizations are both important for promoting EBP use [29]. Thus, we combined a focus on first-level leader development with organizational support in order to optimize efforts to support EBP implementation.

### Leadership and change within an organizational context

Leadership is critical in effective implementation of innovation in organizations in general, and in health care in particular [39,40], but the leader’s actions generally occur within the context of an organization. Congruence of organizational strategies across levels with leadership effectiveness increases the likelihood that organizations will be able to effectively implement and sustain change [9,41,42]. For example, the organizational level (e.g., corporate level) and the unit level (i.e., first level) are both considered important in many implementation frameworks [43,44]. Consistent with the LOCI approach, studies utilizing models such as the Promoting Action Research in Health Services (PARIHS) framework have identified the need to consider context, facilitation, and evidence [45], as well as transformational leadership (a key part of FRL) [46] during implementation. In LOCI, we focused on developing first-level leader foundational leadership skills (i.e., FRL) in order to support strategic leadership where leaders demonstrate readiness and support for interventions with strong research evidence (i.e., EBPs) and their implementation (i.e., implementation leadership). LOCI also emphasizes organizational context and the development of organizational strategies by involving executive management, middle management, and the first-level leaders working together to identify and provide changes in organizational structures and/or processes to support EBP implementation and sustainment [47]. For example, an executive director can send emails to each team member emphasizing the importance of EBP implementation to the mission of the organization and in assuring effective client or patient outcomes. Organizations may bolster fidelity processes or provide recognition or incentives for excellence in EBP delivery. Similarly, middle managers may attend team meetings and support the first-level team leader’s emphasis on utilizing EBP. Thus, LOCI takes a complementary approach of leader development coordinated with the development of practical and ideological support strategies across organization levels to facilitate provider EBP use [48].

### LOCI Intervention development and content

The LOCI development team was comprised of academic researchers with expertise in leadership, organizational climate and culture, health services research, and EBP implementation; a representative from the California Institute for Mental Health; and a community mental health program manager. External consultants brought additional expertise in leadership, implementation, team dynamics, and adult learning curriculum design. In the first year of the project, the team met weekly to identify, define, and adapt the leadership intervention through in-person meetings, email communications, real-time online and in-person review of materials, feedback on materials, and decisions on content and method of delivery. The resulting content of LOCI has six key aspects: 360° assessment (including FRL, implementation leadership, and implementation climate), a 2-day group-based interactive and didactic training session with leadership development planning, weekly coaching, organizational strategy development with the first-level leader and organizational upper and middle management, one in-person group booster session, and graduation. The first 3 months of LOCI focused on developing foundational (i.e., transformational and transactional) leadership behaviors, while the latter 3 months focused on developing strategic leadership and climate for EBP implementation. A detailed description of the LOCI development and training can be found in Additional file 1.

### The present study

As recommended by Leon and colleagues [49] pertaining to the scope of pilot studies, and in line with the NIH funding mechanism supporting this work (i.e., exploratory/developmental grant), the main goal of this pilot study was to assess the feasibility, acceptability, and perceived utility of LOCI. We also assessed preliminary effects of LOCI on supervisee-rated leader readiness and support behaviors. The study design was a mixed method (quantitative/qualitative) two-arm randomized pilot study in which leaders were assigned to LOCI or to a webinar control condition [50]. We predicted that leader participants in LOCI, compared to the control condition, would report higher scores on quantitative measures of feasibility, acceptability, and utility. We predicted that qualitative data would support the feasibility, acceptability, and utility of the LOCI intervention. Finally, we predicted that clinicians supervised by leaders receiving LOCI, compared to those in the control condition, would report higher scores on quantitative measures of Leader Readiness and Support for EBP (i.e., implementation leadership).

## Method

### Recruitment

After receiving institutional review board approvals, recruitment was conducted by first contacting executive management at three community-based mental health organizations in California, USA. All three agencies (100%) agreed to participate. Agency upper and middle managers informed their program leaders that participation in the study was available and they could volunteer to participate. Volunteer program leaders’ names and contact information were provided to the investigative team. After volunteer program leaders were identified and recruited, their clinical staff members were informed about the study in an email, and they were given the opportunity to provide consent and participate or decline through the web-survey interface. Leaders did not know whether or not a given clinician participated or not.

### Participants

Participants were mental health program leaders (*n* = 12) and the clinicians they supervised (*n* = 100). Managers were randomized to the LOCI (*n* = 6) or control condition (*n* = 6). The demographic makeup of the leader sample was 75% female, 58.3% Caucasian, 16.7% Hispanic, 16.7% Asian American, and 8.3% African American. Mean leader participant age was 39.58 years (SD = 8.48; range = 32–62). One manager randomized to the LOCI condition was promoted after the initial training and could no longer participate, and was therefore excluded from analyses.

Data were collected from clinician supervisees in both conditions (LOCI, *n* = 41; control, *n* = 59). Sample size varied between the two conditions because leaders supervised different numbers of clinicians and randomization was at the leader level. For longitudinal analyses, sample size varied at each wave because of staff turnover and replacement; data from supervisees of the one excluded manager were not utilized. The clinician sample was 80.6% female, 46.9% Caucasian, 29.6% Hispanic, 8.2% African American, 7.1% Asian American, 2.0% American Indian, and 6.1% “other.” Mean clinician participant age was 37.6 years (SD = 9.0; range = 26–65) and mean job tenure was 3.58 years at baseline. The educational attainment of the sample was high school or some college (9.2%), college graduate (43.9%), master’s degree (45.9%), or PhD (1.0%). Seven percent were licensed professionals with 62.4% being unlicensed or interns (30.6%). Interns and unlicensed professionals worked under the supervision of a licensed professional.

### LOCI pilot study conditions

Managers were randomized to either the 6-month LOCI intervention condition (didactic training, coaching, and multilevel organizational strategy) or the control condition (two 1-hour leadership webinars focusing on leading change through “Creating a clear and compelling vision” and addressing “Responses to change”). Random assignment was conducted within agency and balanced to minimize those in different conditions being located in the same geographic location, thus decreasing the likelihood of contamination. There were no significant differences between the two groups in the proportion of males and females, Hispanic vs. non-Hispanic race, or education level (*p*s > .05). There was a slightly greater proportion of participants that worked full-time in the control condition (100%) compared to the LOCI condition (89.2%) (*p* < .05) and a small age difference between the control (M = 35.51, SD = 7.73) and LOCI (M = 39.95 years, SD = 10.18; *p* < .05) groups. The lack of differences and a small magnitude of difference in only two variables mitigate concern that the two groups were meaningfully different. Managers in the LOCI intervention condition participated in the LOCI training as described previously. The webinars were provided by a leading research and consulting group on leadership and leader development. Webinars could be completed at a time convenient for each control condition manager within the first month of the study. No follow-up coaching was provided for participants randomized to the control condition. As shown in Additional file 2, the elements of the LOCI and control conditions were mapped onto the Taxonomy of Behavior Change Techniques as identified by Michie et al. [51] in order to enumerate the classes of strategies utilized.

### Measures

Mixed quantitative and qualitative methods were utilized in this pilot study. Table 1 shows the different types of data collected in the study and who reported about each of the principal dimensions of interest (i.e., leader or clinician).

**Table 1:** **Leadership outcome dimensions, method, & data source**

**Outcome dimension**	**Leader report**	**Leader report**	**Supervisee report**
	**Qualitative**	**Quantitative**	**Quantitative**
Feasibility	X	X	
Acceptability	X	X	
Utility	X	X	
Leader support for EBP			X
Leader readiness for EBP			X

### Quantitative methods

Quantitative measures were collected from leaders participating in the study and from their teams of clinicians at baseline (prior to leader training), 3 months, and 6 months after training. Measures developed for this study were reviewed for face validity by the investigative team and external program managers prior to data collection. Data were collected via web-based surveys. Response rates were 100% for leaders and 82% for clinicians across the three waves.

### Leader self-report

Quantitative data from leaders included ten items assessing feasibility, acceptability, and perceived utility of the leadership training (LOCI or control). These items were developed by the research team specifically for this study to assess these three pilot study outcomes [49]. Feasibility was assessed by asking about the degree to which participants were engaged in thinking and learning about leadership and implementation. Example feasibility questions included: “How often did you think about the leadership training?” (0 = not at all to 5 = once an hour) and “How much did you learn from the leadership training?” (0 = nothing to 4 = a very great amount). Acceptability was assessed by asking about the degree to which leaders accepted and applied what they were learning. Example acceptability questions included: “Over the past 6 months, approximately how often did you apply what you learned in the leadership training?” (0 = not at all to 5 = once an hour) and “To what extent did you change your leadership behaviors based on what you learned in the leadership training?” (0 = not at all to 4 = to a very great extent). Perceived utility of LOCI was assessed with questions assessing the degree to which the overall experience was useful. An example utility question was: “To what extent was the leadership training useful in regard to implementing or using evidence-based practice in your team?” (0 = not at all useful to 4 = extremely useful).

### Leader readiness and support for EBP

Items for the leader readiness and support scales were developed by the investigative team as no measures assessing these constructs were available. Item content was developed based on literature identifying aspects of leadership related to a strategic climate for implementation [9] and items were scaled from 0 (not at all) to 4 (to a very great extent). Consistent with psychometric theory, we assumed an underlying continuum for items, and in particular, for aggregate scales [52,53]. A sample Leader Readiness item is “[Supervisor name] has a plan to address implementation of evidence-based practice,” representing clinician perceptions of the leaders’ planning and problem-solving behaviors related to EBP implementation and use (five items, α = 0.89–0.95 across three waves). A sample item for the Leader Support scale was “[Supervisor name] is strongly committed to the successful implementation of evidence-based practices,” representing a leader’s active commitment, advocacy, and actions to support EBP implementation and use (six items, α = 0.79–0.86 across three waves).

### Qualitative methods

Qualitative data were collected from leaders at the initial training session and 3 months later at the booster training session for the LOCI condition, at the conclusion of the webinar sessions for the control condition, and 6 months after the initial training for both groups.

### Open-ended survey questions

Manager participants (*n* = 11; 100% of managers) completed a survey of open-ended questions about the feasibility (e.g., “What were the key things that you learned from the leadership training?”), acceptability (e.g., “Was there anything in the training that you would have changed or done differently?”), and perceived utility (e.g., “Which parts [of the training] did you find particularly useful or not useful?”) of the LOCI or webinar training. For questions where a simple yes/no answer was possible, follow-up questions elicited more detailed responses.

### Focus group

A doctoral-level sociologist conducted a focus group with LOCI condition participants (*n* = 5) following the completion of the training program. A semi-structured focus group guide was used to elicit leader perspectives on the overall project, the initial 2-day training, the follow-up training, weekly coaching, group conference calls, the feedback provided, web surveys, organizational strategies that were developed, and facilitation of multi-level interactions within participants’ organizations (see Additional file 3 for focus group guide).

### Quantitative analyses

Leader self-report data were analyzed using independent groups *t*-tests comparing LOCI vs. control participant responses. As recommended to provide a common metric for interpretability, we also report the effect size for mean differences where a Cohen’s “*d*” of 0.80 or greater represents a large effect [54,55]. Analyses of clinician (i.e., supervisee) report of Leader Readiness and Leader Support scores were conducted utilizing mixed effects (i.e., hierarchical linear) models [56]. These analyses controlled for the fact that clinicians were “nested” in leaders. That is, clinicians at level 1 (individual respondent level) were nested in supervisors at level 2 (workgroup level). There were minimal differences in demographics across the two groups and we did not find differential attrition, or missing data across groups, variables, and waves, reducing concern regarding meeting assumptions (i.e., MAR) for the statistical tests applied. We compared LOCI and control groups on initial intercept (i.e., baseline) and slopes across the three waves using Mplus 7.11 [57] for growth modeling and accounting for the nested data structure and clinician gender. We tested slope differences over the course of the study (controlling for baseline intercept). Thus, the model did assess both intercept and slope; however, intercepts at baseline were not significantly different. The primary issue of interest was change in slopes over time and thus we emphasize that outcome.

### Qualitative analyses

Managers’ responses to open-ended questions were compiled into a single document, and the focus group proceedings were audio-recorded, professionally transcribed, and reviewed for accuracy. Both sets of data were then coded and analyzed using NVivo software [58]. The analyses were undertaken by two research assistants supervised by GAA and the sociologist. The analytic framework as described by Patton [59] focused on the use of sensitizing concepts, which are categories that the analyst brings to the analysis of the data. In this instance, the *a priori* constructs centered on feasibility, acceptability, and perceived utility. In providing a general sense of reference to both data collectors and analysts, sensitizing concepts help guide how data are organized and described.

Analysis proceeded first by engaging in *open coding* to locate key issues pertaining to the feasibility, acceptability, and perceived utility of the LOCI organizational intervention. Segments of text ranging from a phrase to several paragraphs were assigned codes based *a priori* on these three key constructs and the specific questions comprising the surveys and the focus group. During this review of the data, new codes not considered previously were also identified. *Focused coding* was then used to determine which of these issues recurred and which represented unusual or particular concerns to participants regarding LOCI. In this staged approach to analysis, the research assistants drafted memos describing and linking codes to one another [60] and met with GAA and the sociologist to define the inclusion and exclusion criteria for assigning specific codes [61]. This process led to an enhanced definition of codes and resulted in a high level of coding agreement (*r* = 96%). Through the process of constantly comparing and contrasting codes, the investigative team then grouped codes with similar content or meaning into broad themes linked to segments of text in the survey and focus group datasets.

## Results

### Quantitative results

We assessed item and scale distributions for both leader self-report individual items and clinician ratings on the leader readiness and leader support scales. All items and scales were normally distributed with no significant departures related to skewness or kurtosis. In addition, there were no statistically significant differences in variances for any of the leader report items or clinician report scales across the two groups.

### Leader self-report

As shown in Table 2, LOCI participants reported significantly higher feasibility, acceptability, and utility of training compared to those in the control condition. We also conducted chi-square likelihood ratio tests for all single-item variables and results were consistent with those in Table 2. Thus, we report only the parametric results in the table. In regard to feasibility, LOCI participants reported greater engagement in leadership training and a significantly greater gain in knowledge about leadership. In regard to acceptability, LOCI participants reported greater application of what was learned during training, greater leadership improvement, and greater ability to manage change. LOCI participants also reported significantly greater change in behavioral routines, improvement in leadership behaviors, and increased emphasis on EBP in interactions with supervisees. Finally, LOCI participants reported significantly higher overall perceived utility of the training, utility of the training in managing general change and organizational change, and for implementing and/or using EBPs in their teams.

**Table 2:** **Leader report: LOCI and control conditions t-tests for cognitive change, behavioral change, and perceived utility of LOCI**

**Variable**	**LOCI**		**Control**				
	**(** ***n*** **= 5)**		**(** ***n*** **= 6)**				
	**M**	**SD**	**M**	**SD**	***t(*** ***df*** ** = 9)**	***p***	**Cohen’s** ***d***
Feasibility							
Engagement in leadership training	3.20	1.30	1.67	0.82	−2.39	.041	1.45
Increased leadership knowledge	3.40	0.55	1.33	0.52	−6.43	.000	3.89
Acceptability							
Applied learning	3.60	0.55	1.00	1.09	−4.80	.001	2.91
Leadership improvement	3.00	0.71	0.83	0.75	−4.88	.001	2.96
Ability to manage change	2.80	0.84	0.50	0.84	−4.54	.002	2.75
Change behavioral routines	3.20	0.84	0.83	0.75	−4.94	.001	2.99
Changed leadership behaviors	3.20	0.45	1.00	0.89	−4.97	.001	3.01
Increased emphasis on EBP	3.00	1.00	0.83	0.75	−4.11	.003	2.49
Utility							
Utility—general	3.60	0.55	1.00	0.63	−7.20	.000	4.36
Utility for managing org. change	3.00	0.71	0.33	0.82	−5.72	.000	3.46
Utility for implementing EBPs	3.60	0.55	0.83	1.17	−4.84	.001	2.93

All variables were measured on a 0–4 scale except “engagement in leadership training” and “applied learning” which were measured on a 0–5 scale. Cohen’s *d* is an effect size where a value of .80 or greater indicates a large effect.

*M* mean; *SD* standard deviation.

### Clinician (i.e., supervisee) report of leader behavior

**Figure 1:**
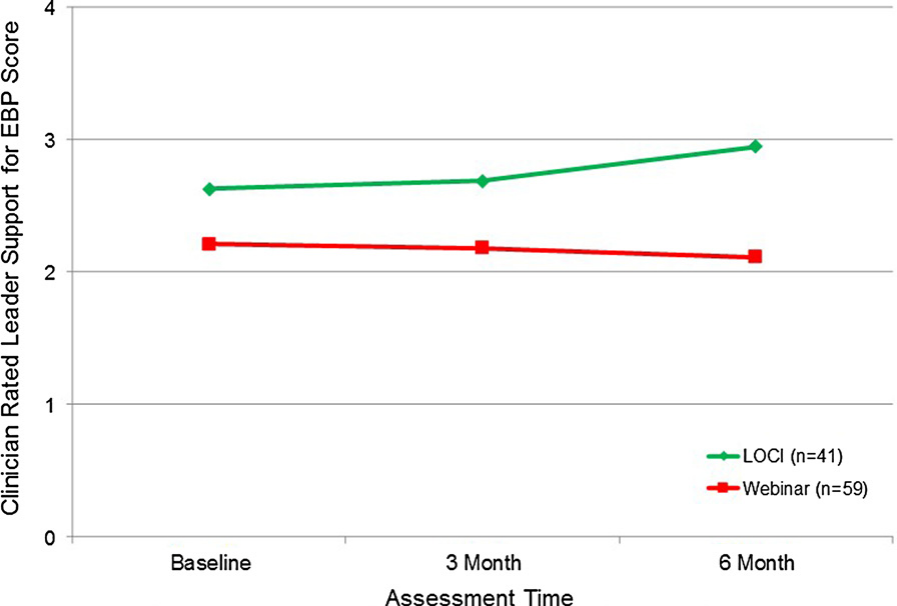
**Supervisee report of leader support for EBP: means over time by condition (** ***p*** **< .05).**

As shown in Table 3, we found no significant effects for Leader Readiness across the three waves or at 3 months or for Leader Support at 3 months. However, as shown in Figure 1, we found a significant slope effect for leader support indicating that the LOCI group support scores increased from baseline to 6-month follow-up relative to the control group (*b* = .248, *p* < .05).

**Table 3:** **Clinician reported leader readiness for EBP and support for EBP scales means, standard deviations, and sample size at each wave, over time by condition**

	**Baseline**			**3 months**			**6 months**		
	**Mean**	**SD**	***n***	**Mean**	**SD**	***n***	**Mean**	**SD**	***n***
Leader readiness for EBP									
LOCI	2.05	0.80	28	2.11	0.94	29	2.18	0.93	23
Control	1.46	0.90	39	1.33	0.99	32	1.53	0.93	33
Leader support for EBP									
LOCI	2.60	0.73	28	2.66	0.73	29	2.98	0.86	23
Control	2.24	0.71	39	2.12	0.62	32	2.16	0.63	33

All variables were measured on a 0–4 scale. The Leader Readiness for EBP and Leader Support for EBP scales met assumptions of normality. The overall reported sample size refers to all of the participants across all three waves. Individuals who do not have all three time-points are included in the analyses; *n* = 100; LOCI *n* = 41, webinar control condition *n* = 59 across three waves.

*SD* standard deviation, *n* sample size by group at each wave.

### Qualitative results

For the qualitative analyses, we focused on the *a priori* constructs of (1) *feasibility, (2) acceptability*, and (3) *perceived utility* of the LOCI organizational intervention to supervisors. We present brief results here, and more detailed qualitative results can be found in Additional file 4.

### Feasibility

The various aspects of the LOCI training (initial training, weekly coaching calls, group conference calls, and booster session) were seen as practical, efficient, realistic, and even desirable. The issues related to feasibility involved the fit with job responsibilities and work constraints, the efficiency of the in-person training, flexibility of training and coaching, and survey burden (for 360° assessments for clinicians). For example, there was concern with the length of the clinician survey for multiple assessments.

### Acceptability

LOCI was viewed positively by participants in regard to the leadership approach, development of clear training goals subject to revision based on data and experience, safety and trust within the training group, relevance to day-to-day work, and personal growth. Participants reported that they experienced the didactic presentation format and content as engaging and that both the conceptual and visual content were helpful. The brief weekly coaching calls were perceived as meaningful and helpful in keeping participants focused on leadership skills and goals and facilitated problem-solving in emergent leadership issues such as garnering buy-in and support from middle management. Social support from in-person trainings and monthly group conference calls allowed participants to share ideas and gain insight from one another’s successes and challenges. The LOCI team was characterized as accessible and enthusiastic, which facilitated engagement and participation. The primary concern with regard to acceptability was that participants desired more support from the LOCI team in navigating multiple concerns and responsibilities in the face of competing work demands, such as juggling productivity while supporting quality service provision. In contrast to the LOCI intervention, control participants noted that the webinar format was too simplistic, not engaging, and lacked interactive learning processes. Control participants also expressed a perceived disconnect between the material presented and being able to remember and apply the learning to their work contexts. For example, one of the webinars targeted employee responses to organizational change but was more focused on general organizational change rather than change related to implementation of EBP.

### Utility

LOCI was perceived as being useful and helpful in day-to-day operations and in implementing general changes (e.g., work routines) and EBP. The coaching was seen as useful for staying on track in contrast to other didactic only trainings that did not provide follow up. The FRL model was seen as applicable and useful for allowing leaders to understand their own leadership approach and to encourage positive staff attitudes toward EBP. LOCI was described as an important tool for EBP implementation and use and relevant to apply in the allied health service settings in which participants worked. LOCI was also seen as helpful in upward influence on middle and upper management in the organization. Participants suggested that the feedback from the 360° assessments made it possible to recognize personal leadership strengths and weaknesses and to collaborate with the LOCI team to create individualized personal development plans that were then the subject of coaching. The LOCI trainees also reported utilizing what was learned in training to encourage and support staff in the use of EBPs. Moreover, the training provided a sense that they could accomplish change. Participants also expressed some concerns about the intervention, especially in regard to lack of time to apply all components of LOCI.

### Integration of mixed methods

As shown in Table 4, both quantitative and qualitative results supported the feasibility, acceptability, and utility of the LOCI intervention. In most cases, we found convergence across methods, and in others, additional information provided expansion of findings [62,63]. For example, in examining convergence of findings, quantitative and qualitative results generally supported feasibility. However, the expansion of the findings was evident in the issue of the length of assessments being identified as a potential feasibility hurdle in the qualitative but not the quantitative analyses. The analyses converged in supporting acceptability; however, the content of quantitative (e.g., application of learning, change in behavior) and qualitative (e.g., acceptance of the FRL model, relevance to day-to-day work) results provided expansion of findings. Results regarding utility were generally convergent across methods.

**Table 4:** **Integration of mixed method results demonstrating convergence and expansion of findings**

**Approach**	**Quantitative**	**Qualitative**
Question	Is the LOCI intervention feasible?	
Answer	Yes: Leaders in the LOCI condition reported being more engaged in the training and learning more than control condition participants	Yes: LOCI participants were able to articulate more comprehensively the aspects of training that were important for EBP implementation.
		Yes: The LOCI training, including initial training, coaching, group calls and booster session were seen as feasible and desirable even in the face of competing demands.
		No: Participants in both conditions noted that staff surveys were too long
Question	Is the LOCI intervention acceptable?	
Answer	Yes: Leaders in the LOCI condition compared to the control reported applying what was learned, ability to manage change, change in behavioral routines and leader behaviors and an increased emphasis on EBP in their interactions with supervisees to a greater degree	Yes: LOCI was acceptable in regard to the FRL conceptual model, use of specific and measureable training goals, relevance to day-to-day work, and personal growth.
Question	Does the LOCI intervention have utility for evidence-based practice implementation?	
Answer	Yes: Leaders in the LOCI condition, compared to the control, reported greater general utility, utility for managing organizational change, and utility for EBP implementation.	Yes: LOCI participants reported that the intervention had utility in day-to-day operations, implementing general change, and implementing change related to EBP
	Yes: Supervisees of leaders in the LOCI condition, compared to the control condition, reported increased leader support for EBP	

*FRL* Full-Range Leadership.

## Discussion

The main finding of this pilot study is that the LOCI organizational intervention was judged to be feasible and acceptable and to have utility for developing leaders with the potential to support EBP implementation in organizations. The study also showed clinician-rated change in leader behavior. Overall, the LOCI intervention was seen as positive, balanced in its approach, and accessible and supportive at the leader and organizational levels, characteristics likely to enhance the probability that the LOCI strategy can be utilized by organizations implementing EBPs [64].

The LOCI intervention utilized the FRL model as a foundational theoretical approach to facilitate leader readiness and support for EBP and LOCI and incorporates training specific to leading and overcoming hurdles to EBP implementation [38]. For example, consistent with findings from other studies, LOCI promotes leaders being proactive and present while increasing leaders’ knowledge of various EBPs to address health issues in their particular setting [29]. LOCI also focuses on applying individualized consideration to aid in demonstrating support for EBP. LOCI has at its roots a problem-solving orientation in which leaders persevere through the ups and downs of the implementation process [38,65]. LOCI also promotes key leadership behaviors consistent with other approaches such as creating a shared vision and demonstrating behaviors that followers will seek to emulate [66]. While there have been some criticisms regarding charismatic leadership models such as the FRL [67], they remain important for understanding and improving organizational processes and appear to have utility for EBP implementation.

Leader Readiness for EBP was not significant in our analyses. It may have been that “readiness” is less observable and more difficult to demonstrate than more overt behaviors. Alternatively, it may take more than 6 months develop and demonstrate “readiness” to a degree that it will be recognized by supervisees. Additionally, readiness focuses on preparation for a new implementation and leaders may not have had the opportunity to demonstrate readiness for EBP if their team was later in the process of implementation. Refinement of the construct of “implementation leadership” continued after the present study and a new brief measure of implementation leadership holds promise to advance future research [38].

This study suggests a need for an ongoing focus on how to apply general leadership behaviors [68] while also focusing on strategic implementation leadership and climate. For example, FRL and strategic implementation leadership training could be better integrated with the development of implementation climate (e.g., using transformational leadership to motivate staff regarding implementation). This would support leaders in maintaining FRL behaviors while utilizing implementation leader skills and behaviors, and simultaneously developing strategic climates for implementation [9].

One key feature of LOCI is the combination of first-level leader development and organizational strategies for improved implementation. This approach of individual development in the context of organizational development and change is one that has the potential to capitalize on both individual and organizational strengths [69] and may enhance generalization to other types of organizational development initiatives in other settings [70]. Future work in this area should assess the degree to which more or less formalization of the organizational development component of LOCI leads to greater change in leadership effectiveness and organizational context. Previous studies have found that organizational development interventions can improve workplace climate and patient-level outcomes [71]. Future studies should examine the extent to which strategic climates can be developed to support EBP implementation [72].

### Limitations

Some limitations of this study should be considered. First, the sample size was not large and this may have impacted our ability to find significant effects for some measures. This was, however, a pilot study focused on feasibility, acceptability, and utility, and results provided encouragement for moving forward to further test this empirically derived implementation strategy. Second, some of our outcomes relied on leader self-report. Leaders were randomized to conditions and this should help to equalize potential for reporting bias. Additionally, the results for this study are from multiple perspectives as subordinate ratings of leader behavior were also assessed. We were able to examine leaders’ assessments of how much they were using what they learned in LOCI and how much change there was in the behaviors from both the leaders own and their supervisees’ perspectives. Third, there may be discrepancies in supervisor vs. clinician report of leader behavior. While such discrepancies may be associated with organizational characteristics [73], the small sample size in this study precludes a viable examination of this issue here. Future studies should examine the potential role of discrepancy in implementation leadership ratings and associations with organizational functioning during implementation and sustainment. Fourth, the clinical teams that participated were at various stages of EBP implementation and sustainment. Because this was a pilot study, it was not possible to arrange for all teams to be implementing the same intervention at the same time. Future studies should examine the effectiveness of LOCI in facilitating leader development, organizational change, and implementation effectiveness and outcomes [74]. Fifth, assessment of feasibility, acceptability, and utility did not focus on specific strategies that were part of individual leader development plans. Future studies should more clearly identify and assess such strategies. Finally, the LOCI intervention was only 6 months in length. The supervisee reports of some leader behaviors showed that the rate of change increased over the course of 6 months (i.e., support). This finding, together with the qualitative results, suggests that a longer time may be needed to achieve desired effects. Future studies should test LOCI for longer periods of time and follow up on a more diverse set of outcomes.

### Strengths

A number of strengths of the present study should be noted. LOCI is an outgrowth of an empirically valid and supported theoretical approach and practical framework for leadership development and unit level change [75,76]. Our process for LOCI development included stakeholders from multiple organizational levels including direct service providers, program supervisors, executive directors, and experts in leadership and implementation science. This facilitated initial development of LOCI. We obtained data from both self-report and staff-report measures to obtain a multi-perspective view of the LOCI training. We utilized quantitative and qualitative data to assess the feasibility, acceptability, and utility of the LOCI training. Finally, this study took place in a mental health services setting, a growing area represented in the allied health implementation literature.

While these preliminary results regarding LOCI were generally positive, some issues and recommendations for improvements were made by participants and researchers. First, there was a need to increase early involvement at the middle and upper organizational levels to support initiatives being spearheaded by first-level leaders. To this end, we have instituted a schedule of multilevel organizational strategy development meetings beginning at the inception of LOCI training to make these activities consistent with broader organizational goals and initiatives [77]. Another concern was the amount of time it took staff and managers to complete the survey assessments due to their length. Thus, to increase feasibility, we have shortened the 360° assessment by 40% and included new brief measures of implementation leadership [38] and implementation climate [78]. The result is a more streamlined and targeted assessment that should facilitate more effective deployment of LOCI.

## Conclusions

The present study provides support for the development and deployment of active strategies to improve EBP implementation in health and allied healthcare organizations. Although leadership in general has been shown to support effective implementation [23,28-31,79], the LOCI intervention highlights specific strategies that leaders can use to improve the climate for implementation in their teams. Efforts that do not consider both contextual and individual factors likely to facilitate or hinder EBP implementation may result in substandard service delivery, compromised client outcomes, and decreased public health impact. Strategies that assess, intervene, and support implementation at multiple organizational levels should have a greater likelihood of success in the effective deployment of EBPs. Such a complementary approach should lead to improved EBP implementation, sustainment, and public health impact.

## Additional files

Additional file 1:
**Detailed description of the LOCI development process.**
Additional file 2:
**Elements of the LOCI and control conditions placed within the Behavior Change Taxonomy (BCT).**
Additional file 3:
**LOCI Focus group guide.** Qualitative Guide for Post-LOCI Meeting with In-Person Participants. Additional file 4:
**LOCI Detailed qualitative results.**
