# Supplementary material for: Leadership and organizational change for implementation (LOCI): a randomized mixed method pilot study of a leadership and organization development intervention for evidence-based practice implementation
Source: Implement Sci. 2015 Jan 16;10:11. doi: 10.1186/s13012-014-0192-y (PMC4310135; doi:10.1186/s13012-014-0192-y)
Supplement: Additional file 1: — Detailed description of the LOCI development process. [file 13012_2014_192_MOESM1_ESM.pdf]

ADDITIONAL FILE 1:

DETAILED DESCRIPTION OF THE LEADERSHIP AND ORGANIZATIONAL CHANGE FOR IMPLEMENTATION (LOCI) DEVELOPMENT PROCESS

Leadership and organizational change for implementation (LOCI): A randomized mixed-method pilot study of a leadership and organization development intervention for evidence-based practice implementation

Gregory A. Aarons[1,2 §], Mark G. Ehrhart[3], Lauren R. Farahnak[1,2], Michael Hurlburt[2,4]

[1]Department of Psychiatry, University of California, San Diego, La Jolla, CA USA
[2]Child and Adolescent Services Research Center, San Diego, CA USA
[3]Department of Psychology, San Diego State University, San Diego, CA USA
[4]School of Social Work, University of Southern California, Los Angeles, CA USA

[§]Corresponding Author

DETAILED DESCRIPTION OF THE LEADERSHIP AND ORGANIZATIONAL CHANGE FOR IMPLEMENTATION (LOCI) DEVELOPMENT PROCESS

LOCI Intervention Development

The core LOCI development team was comprised of academic researchers with expertise in leadership, organizational climate and culture, mental health services, and EBP implementation, a representative of the California Institute for Mental Health, and a community mental health program manager. For some of the meetings a curriculum development consultant also joined the investigative team to provide input on formatting and packaging training materials. External project consultants brought additional expertise in leadership, implementation, and teams. In the first year of the project, the team met weekly to identify, define, and adapt the leadership intervention through in-person meetings, email communications, real-time online and in-person review of materials, feedback on materials, and decisions on content and method of delivery.

LOCI Development Process. Weekly meetings were utilized to work on curriculum development and strategic planning. The team identified the FRL model [1] as the most applicable validated and theory-driven leadership curriculum to tailor for the LOCI intervention. One of the developers of the FRL model (B. Avolio) consulted with the team by conference call and also met with the investigative team in person to provide consultation on leadership intervention development and to work on tailoring the leadership training plan for EBP implementation. The team continued an iterative and ongoing process of adapting and tailoring the curriculum for EBP implementation. The team also developed a structure for the development of individualized organizational strategies to support leadership and organizational change.

A meeting of participating agency administrators was convened in July 2009 to introduce the project and ask for their feedback on the curriculum. Additionally, a group of local mental health service program managers met to form a "Program Manager Advisory Group." These meetings supported a collaborative process in order to adapt and develop LOCI with the goal of refining the intervention and delivery process to fit the needs and preferences of mental health service agency administrators and program managers. All feedback was considered and collective decisions were made with regard to the content and process of the LOCI intervention.

The investigative team also met with agencies to recruit mental health program leaders for participation in the study. In these meetings, the team discussed the potential benefits and logistics of participating in the LOCI project. Potential participant agency directors were told about the length of the intervention, web surveys for data collection and organizational feedback, and the randomization process that would occur within each agency. Interested agencies suggested leaders that would be eligible to participate as trainees based on availability and level

within the agency.

LOCI Training Content and Process

The LOCI intervention aims to improve organizational leadership and organizational context for supporting implementation of EBP during various phases of implementation identified in the EPIS implementation framework that considers outer system and inner organizational contexts through the phases of Exploration (i.e., consideration of EBP to implement and fit with system and organization), Preparation (i.e., planning activities for implementation) , Implementation (active implementation such as training and coaching in the EBP), and Sustainment (i.e., continued use of an EBP with fidelity) as well as outer (system) and inner (organization) contexts [2]. However, this study focused the inner organizational context in order to develop targeted and individualized leader development plans and organizational strategies that meet the needs of a given organization and leaders within that organization. LOCI is multilevel in nature, engaging stakeholders at the executive, mid-management, and first-level leadership levels, in order to create a context in which first-level leaders are supported to maximize their leadership capacity and effectiveness. As noted above, first-level leaders are those who supervise employees who do not supervise others and this includes the many first-level leaders who often directly supervise clinical or case-management staff in health and allied health settings [3].

In addition to facilitating development of organizational strategies for supporting effective EBP implementation, the LOCI training includes individualized leadership development plans and weekly coaching calls that support learning and behavior change of first-level leaders who are considered the main local change agents in the LOCI approach. We integrate Stoltenberg’s developmental model of supervision in the coaching of first-level leaders

and in training them to interact effectively with their own staff [4]. Thus, LOCI could be integrated across implementation phases and levels but should be most useful in the preparation and implementation phases. In addition, LOCI should be scalable depending on organization or system size.

LOCI consists of seven specific components: 1) Assessment, 2) Didactic Training, 3) Leadership Development Planning, 4) Coaching, 5) Organizational Strategy Development; 6) Booster Session, and 7) Graduation. We describe each of these components below.

1. Assessment: LOCI is a data-driven process with leader development plans and goals based on 360 degree assessment with measures completed by the leader for a self-assessment, by each person supervised by the leader, and by the leader’s supervisor. The data are synthesized into a detailed feedback report and then used in the development of a personal development plan.

2. Didactic Training: The LOCI program training then begins with a two-day leadership didactic and interactive session. Day one includes group introductions, introduction to the full-range leadership model, identifying transformational and transactional leadership behaviors, identifying how full range behaviors can be used to build a climate for EBP implementation, and group activities (e.g., breakout groups, meals) to facilitate social interaction and learning consolidation among trainees. The didactic portion of day two addresses understanding the nature of EBPs so that leaders can then articulate a rationale for how and why EBPs can improve patient and client outcomes.

3. Leadership Development Planning: The second half of day two involves leadership

development planning and involves trainers working individually with each trainee in reviewing their personalized 360 degree assessment data, identifying strengths and areas for development, and setting a timeline for issues to be addressed immediately and those to be addressed later in the coaching. Leaders emerge with data-based development plans including broad goals and specific action items that will guide coaching sessions throughout the remainder of the program. However, development plans could be modified based on goal attainment or emergent issues or needs.

4. Coaching: Weekly coaching calls were provided for each LOCI participant. Coaching calls ranged from 15-30 minutes in duration and were intended to keep participants on track with their goals and development plans. For the pilot study, the coach was a Ph.D. level psychologist who had been part of the LOCI development team, was well versed in the FRL leadership model, expert in the delivery of clinical services, and who worked with participants in developing their individualized plans. The weekly coaching calls focused on tracking the trainees' progress in their development plans, problem solving, providing additional leadership support, and identifying organizational strategy needs throughout the duration of the intervention. Plan-Do-Study-Act (PDSA) cycles were utilized to assess incremental progress toward measureable overall and time-limited goals [5, 6]. GAA observed a sample of coaching calls and provided feedback to the coach regarding process, content, and approach of the coaching. Group conference calls were also held on a monthly basis to facilitate problem solving and networking among LOCI participants. During these calls trainees discussed their progress and generated solutions to barriers to achieving the goals in their development plans.

5. Organizational Strategy Development: LOCI facilitators met concurrently with executive managers, middle managers, and the LOCI trainees to tailor an organizational strategy to support the leader in their efforts to demonstrate support for and readiness for EBP. Examples of strategies that came out of these meetings included having mid-level managers attend team meetings in support of the first-level leaders, providing recognition for clinical service providers who exemplified excellence in EBP use, providing additional training for EBP when desired or needed, and executives sending out emails emphasizing the importance of EBP in mental health care.

6. Booster Session: Participants attended a booster session three months after the initial didactic training. Leadership principles, intervention goals, and organizational strategies to support leadership were reinforced through group discussion and problem-solving. We conducted additional 360 degree assessments with leaders' supervisees and supervisors prior to the booster session. The booster session was also considered a longer term PDSA cycle in which 360 degree assessments were utilized to assess progress and guide modification of the leaders' development plans. The data were also used as a metric for leaders to assess change over time and see whether supervisee and supervisor perceptions of their own leader behaviors were changing.

7. Graduation: Graduation is a ritual included in LOCI to mark completion of the program. In the pilot study, graduation was held six months after the initial training. During the final graduation meeting, accomplishments of the participants were celebrated. The group also debriefed and discussed successes and challenges in the process. The LOCI team provided feedback from the participants' supervisees and supervisors from baseline, three-month, and six-

month 360 degree assessments. After this meeting, the investigative team also provided aggregate organizational feedback to executive management at each participating agency.

References

1. Bass BM, Avolio BJ: **Training Full Range Leadership.** In *Book Training Full Range Leadership*. City: Mind Garden, Inc.; 1999.

2. Aarons GA, Hurlburt M, Horwitz SM: **Advancing a conceptual model of evidence-based practice implementation in public service sectors.** *Adm Policy Ment Hlth* 2011, **38:**4-23.

3. Priestland A, Hanig R: **Developing first-level leaders.** *Harv Bus Rev* 2005, **83:**112-120.

4. Stoltenberg CD: **Developmental approaches to supervision.** In *Casebook for clinical supervision: A competency-based approach.* Edited by Falender CA, Shafranske EP. Washington, DC: American Psychological Association; 2008

5. Deming WE: *The new economics for industry, government, and education.* Cambridge, MA: The MIT Press; 2000.

6. Langley GL, Nolan KM, Nolan TW, Norman CL, Provost LP: *The improvement guide: A practical approach to enhancing organizational performance.* 2nd edn. San Francisco: Jossey-Bass Publishers; 2009.
