# Supplementary material for: Leadership and organizational change for implementation (LOCI): a randomized mixed method pilot study of a leadership and organization development intervention for evidence-based practice implementation
Source: Implement Sci. 2015 Jan 16;10:11. doi: 10.1186/s13012-014-0192-y (PMC4310135; doi:10.1186/s13012-014-0192-y)
Supplement: Additional file 2: — Elements of the LOCI and control conditions placed within the Behavior Change Taxonomy (BCT). [file 13012_2014_192_MOESM2_ESM.pdf]

ADDITIONAL FILE 2 ELEMENTS OF THE LOCI AND CONTROL CONDITIONS PLACED WITHIN THE BEHAVIOR CHANGE TAXONOMY (BCT).

LOCI Implementation Strategy and Control Condition Elements Placed Within the Behavior Change Taxonomy (BCT)

	BCT Element	LOCI Strategy	Control
1	Scheduled consequences	N/A	N/A
2	Reward and threat	Social reward (in peer and organization recognition of effective leadership)	N/A
3	Repetition and substitution	Habit formation; Habit reversal; Behavioral rehearsal/practice (enacted and reinforced through coaching)	Habit formation; Behavioral rehearsal/practice
4	Antecedents	Restructuring the social environment (tailored to each LOCI participant)	N/A
5	Associations	Prompts/cues (utilized as part of coaching)	N/A
6	Covert learning	N/A	N/A
7	Natural consequences	Social and environmental consequences (as part of learning process)	N/A
8	Feedback and monitoring	Feedback on behavior; Self-monitoring of behavior	Self-monitoring of behavior
9	Goals and planning	Goals and planning, Action planning, Problem solving, Goal setting (outcome); Goal setting (behavior), Review behavior goals, Review outcome goals	Action planning; Discrepancy between current behavior and goal;<br>Goal setting (outcome)
10	Social support	Social support practical and general (through group process)	N/A
11	Comparison of behavior	Modeling of the behavior; Social comparison	Modeling of the behavior
12	Self-belief	Focus on past success; Verbal persuasion to boost self-efficacy	Mental rehearsal of successful performance; Verbal persuasion to boost self-efficacy
13	Comparison of outcomes	Persuasive argument; Pros and cons	N/A
14	Identity	Identification of self as role model; Cognitive dissonance	Identification of self as role model
15	Shaping knowledge	Behavioral experiments, Instruction on how to perform a behavior	Behavioral experiments, Instruction on how to perform a behavior
16	Regulation	Regulate negative emotions; Conserving mental resources	N/A

Note: BCT adapted from: Michie, S., Richardson, M., Johnston, M., Abraham, C., Francis, J., Hardeman, W.,... & Wood, C. E. (2013). The behavior change technique taxonomy (v1) of 93 hierarchically clustered techniques: Building an international consensus for the reporting of behavior change interventions. *Annals of Behavioral Medicine*, *46*(1), 81-95.
