# Supplementary material for: Leadership and organizational change for implementation (LOCI): a randomized mixed method pilot study of a leadership and organization development intervention for evidence-based practice implementation
Source: Implement Sci. 2015 Jan 16;10:11. doi: 10.1186/s13012-014-0192-y (PMC4310135; doi:10.1186/s13012-014-0192-y)
Supplement: Additional file 3: — LOCI Focus group guide. Qualitative Guide for Post-LOCI Meeting with In-Person Participants. [file 13012_2014_192_MOESM3_ESM.pdf]

Additional File 3 LOCI focus group guide.

Qualitative Guide for Post-LOCI Meeting with In-Person Participants

Focus Group with In-Person Participants

Introductions, description of goal of holding this particular focus group, focus group guidelines (e.g., everyone encouraged to participate; divergent opinions and questions welcome), confidentiality, audiotaping

1. We would like to start by hearing about your overall impressions of your involvement with the LOCI project.

    What was good and what was not so good? (probe if necessary)

2. We would like to get your impressions of the initial 2-day training in February. In particular we'd like to get your feedback on the structure, content, and presentation style of that training.

    What did you think about the structure of the training (i.e., lecture component, break-out sessions for individual feedback and formulation of goals on leadership)?

    What did you think about the content of the training (i.e., focus on the full-range leadership model, the overview on EBP, on the different dimensions of implementation climate covered)?

    What did you think about the presentation style of each of the speakers and trainers?

    - Dr. Todd Sosna (EBP speaker)
    - Dr. Bruce Chorpita (PracticeWise speaker)
    - Dr. Greg Aarons
    - Dr. Jonathan Horowitz

    What were your impressions of the audio-visual materials that were used in the training, including the PowerPoint presentations and video clips?

    For each category, probe if necessary: Anything in particular you liked? Anything that you did not like? Do you have any recommendations for things that could be done differently in future trainings?

3. We would also like to get your impressions of the follow-up 1-day training in May.

    What did you think about the structure of that training (i.e., group discussion on leadership model, break-out sessions for individual feedback and formulation of goals on implementation climate)?

    What did you think about the content of the training (i.e., focus on the full-range leadership model, on the different dimensions of implementation climate covered)?

    What did you think about the presentation style of each of the trainers?

    - Dr. Greg Aarons
    - Dr. Jonathan Horowitz

   For each category, probe if necessary: Anything in particular you liked? Anything that you did not like? Do you have any recommendations for things that could be done differently in future trainings?

4. Please describe your experiences with the weekly coaching calls. What was good and not so good?

    If yes, what about those calls was useful and why?

    If no, why do you think the calls were not useful? What might have helped make those coaching calls useful to you? Do you have any recommendations for things that could be done differently in future trainings?

    How was the frequency of those calls? Too frequent? Not frequent enough?

    In general were the topics of those calls useful to you? What were some of the more useful topics? What were some of the least useful topics?

    Occasionally, Dr. Aarons would participate in coaching calls with you and Jonathan. How did this affect the dynamics of the calls, if at all? Would you rather Dr. Aarons had participated more frequently, less frequently?

5. We would like to hear about your experiences with the monthly group conference calls.

    Did you find them useful? Why or why not? Is there anything that could be done to make those group calls more useful?

6. Was it useful to receive feedback on your leadership and on your agency's implementation climate at each of the trainings and at today's meeting? Why or why not?

    How would you rate the format of the feedback (graphs and charts)? Anything in particular you liked? Did not like?

    How would you rate the presentation of the feedback by Dr. Aarons and Dr. Horowitz? Anything you liked in particular? Anything you did not like?

7. We would like to get your feedback on the web surveys. How accurately do you think they measured leadership and implementation climate? Was there anything in particular you liked? Anything in particular you would like to see changed about these surveys?

    Did you get any feedback from your staff about the web surveys? If yes, what kind of feedback did you receive?

8. As a part of the LOCI training, the research team held a meeting with you and others in your organizations about each agency's implementation climate. They also periodically kept agency executives informed on the progress of the project. What do you think about the LOCI team involvement with your organization?

    Was there enough involvement with others in your agency?

    What did you think about the timing of the meeting? Do you feel that we should have met with others in your agencies earlier?

    Did they target the right people, or do you think it would have been helpful to involve different or additional people?

    How comfortable did you feel interacting with other people during the meeting? Was there anything in particular that made you uncomfortable? Would you have liked anything to go differently?

    Did this meeting have any effect on communication about EBPs between you and your supervisors/agency directors?
