# Supplementary material for: Leadership and organizational change for implementation (LOCI): a randomized mixed method pilot study of a leadership and organization development intervention for evidence-based practice implementation
Source: Implement Sci. 2015 Jan 16;10:11. doi: 10.1186/s13012-014-0192-y (PMC4310135; doi:10.1186/s13012-014-0192-y)
Supplement: Additional file 4: — LOCI Detailed qualitative results. [file 13012_2014_192_MOESM4_ESM.pdf]

ADDITIONAL FILE 4: LOCI DETAILED QUALITATIVE RESULTS

Leadership and organizational change for implementation (LOCI): A randomized mixed-method pilot study of a leadership and organization development intervention for evidence-based practice implementation

Gregory A. Aarons[1,2 §], Mark G. Ehrhart[3], Lauren R. Farahnak[1,2], Michael Hurlburt[2,4]

[1]Department of Psychiatry, University of California, San Diego, La Jolla, CA USA
[2]Child and Adolescent Services Research Center, San Diego, CA USA
[3]Department of Psychology, San Diego State University, San Diego, CA USA
[4]School of Social Work, University of Southern California, Los Angeles, CA USA

[§]Corresponding Author

Detailed Qualitative Results

Qualitative Results

For the qualitative analyses we focused on three main questions pertaining to LOCI: Feasibility, Acceptability, and Perceived Utility.

Feasibility. Our question here was whether the LOCI intervention was perceived as feasible in that it could be efficiently conducted with program managers and public sector agencies. The various aspects of the LOCI training (initial training, weekly coaching calls, group conference calls, and booster session) were seen as feasible and even desirable in most cases. The major themes involved fit with job responsibilities and work constraints, the brief in-person training, flexibility of training and coaching, and survey burden (for 360 degree assessments).

Although two days had to be taken away from usual responsibilities for participation in LOCI, the format was seen by most participants as feasible as and better than breaking up the training. In addition, participants noted that the separation from their offices was helpful in providing distance from the distraction of usual activities, allowing for more engagement in the training. For example, leaders stated the following:

“To break it up even though it may be convenient...would really actually

disrupt the flow of learning"

"The overall amount of time was just right… it is easier to disconnect when you're so far out of town."

In the initial planning, the LOCI team considered the work constraints for participants and tailored the didactic training and coaching to be sensitive to those needs. While some participants mentioned difficulty in being away from the office, overall the benefits were perceived to outweigh the costs. The LOCI approach and pacing of activities was also seen as feasible. For example, participants reported the following:

"The time commitment was just right…as far as the number of trainings and the amount of information provided in the trainings and the weekly consults."

The brief and flexible nature of the coaching was seen as contributing to feasibility. The amount of time required (15-30 minutes per week) was seen as appropriate and not overly burdensome.

One of the common complaints of any training is that what is learned often stays in the classroom. LOCI was designed to promote learning and practice of new knowledge and skills. This was reflected in participants' past experiences with trainings, for which it was "very easy just to go and throw your binder on the desk and that's it." The LOCI participants' positive experience of having ongoing coaching with specific individualized goals and accountability is reflected in the following statements:

"I think what really helped was the consistency to know that every week there was going to be that call…going one by one and okay …how are you doing on this [goal]? How are you doing? What are you doing about this [goal]?" and "Just checking in…really kept me on track."

The primary negative feedback on feasibility had to with the staff and participant surveys being too long, but participants also recognized that being comprehensive might be required for the research part of the study. The webinar group made no positive comments regarding feasibility and concerns were related to the time commitment in completing the research survey. For example comments noted that: "Time was provided to participate so that was in addition to an already over-filled schedule. I would have liked to feel that I could spend a little more time with it."

Acceptability. Our question in this domain was if LOCI was acceptable to participants and organizations. We found that overall, LOCI was acceptable in regard to the following areas: the FRL model approach to leadership, the use of specific training goals that were subject to revision based on data, safety and trust within the training group, the relevance to day-to-day work, and personal growth. Data indicated that the didactic presentation format and content were acceptable and engaging, and that the conceptual and visual content was worthwhile. Results also indicated that the training format was directly relevant to leaders' day-to-day leadership obligations and responsibilities. For example, participants noted:

> "Being a visual learner, having the visual and the numerical results [of the 360 assessment] was really key…and motivated me in seeing the different points in my progress. It really was… a motivation to see where I could grow even further."

The brief weekly coaching calls were seen as acceptable, meaningful, and helpful in keeping participants focused, and facilitative for problem solving in emergent leadership issues. For example, on participant stated:

> "I enjoyed the weekly contact with [my coach]…The conversations were

> rich, informative, validating, and motivating… it was nice when [GAA] was involved, also."

In addition, the social support from the in-person trainings and monthly group conference calls allowed for sharing ideas and gaining insight from one another's successes and challenges. The LOCI team was also described as being accessible and enthusiastic, which facilitated engagement and participation.

The primary concern with regard to acceptability was that participants needed more support from the LOCI team navigating the multiple concerns and activities in the face of competing work demands. For example, one trainee noted:

> "For me it was very overwhelming because I only meet with my staff once a week for two hours so here I am trying to [have] a speaker go in…the director needed to send email, there were just so many things."

In contrast to the LOCI group, webinar participants noted that the format was too simplistic, not engaging, and lacked interactive learning processes. As one participant noted,

> "I found the process of learning through the internet to be difficult. Overall I felt this made the entire training uninteresting. I definitely prefer in-person trainings."

There was also a perceived disconnect between the material presented and being able to remember and apply the learning to their work context. Another participant stated:

> "I remember when I watched the webinars thinking they were helpful, but given my schedule, I have had a hard time going back and reviewing."

In addition, some webinar respondents noted that because they were "visual learners" it was difficult to retain the information presented.

Utility. With regard to utility, our question was whether LOCI was seen as being useful and helpful in day-to-day operations and in implementing change and EBP. We found perceptions of utility that related to various aspects of LOCI. The coaching was seen as having high utility; for example, one participant commented:

> "It kept me on track. So to be able to sit down in my office and shut the door and know that every week at 10 o'clock on Thursdays I had a conversation with [my coach] about what the goal was [and] how to get there."

The FRL model was seen as applicable and useful for allowing them to understand their own leadership approach and to encourage positive staff attitudes toward EBP. For example, one participant stated:

> "Not only has the training been applicable for my current position, but also just professional and personally" and "I've been able to utilize the information right out of the gate right away."

In addition, LOCI was seen as having utility for EBP implementation and use: "I was able to… really keep the focus on EBPs." In contrast, there was less support for the utility of the webinar control condition training. One participant noted that "I did not apply what I learned." However, another did state that they applied what they learned in staff meetings.

One of the concerns addressed during the pilot trial was increasing engagement of middle and upper management earlier on in the process, and LOCI was seen as helpful in upward influence to the middle and upper management in the organization. There was a concern, however, in regard to lack of support or time to utilize all components of the LOCI organizational and leadership intervention. Feedback from the 360 degree assessments was seen as having utility for recognizing personal leadership strengths and weaknesses and working with

the LOCI team to create individualized personal development plans that were then the subject of coaching. The LOCI trainees also reported utilizing what was learned in training to encourage and support staff in the use of EBPs.

Overall Findings. Finally, given the opportunity to note what was learned in training, webinar trainees could articulate only 9 aspects of training, whereas LOCI trainees articulated 28 aspects of the training that were important. For example, when asked what key things were learned from the leadership training, webinar participants reported poor recall of what was taught, with one stating that: "To be honest, I am having difficulty remembering the webinars." LOCI trainees reported that they better understood how to apply leadership in supporting EBP and "understanding of the science behind evidence-based programs," and the importance of ongoing coaching in "doing something about [EBP] with the support of the weekly calls by LOCI." LOCI also gave trainees a sense that they could accomplish change. For example, one trainee stated that "change is possible and that little by little you can begin influencing others around you [to] increase the usage of EBP."
